# Editorial: Behavior-Driven Changes in Gene Expression

**DOI:** 10.3389/fnbeh.2022.839395

**Published:** 2022-03-03

**Authors:** Alberto Ferrús, Francisco A. Martin, Luis M. Tuesta, Alfonso Martín-Peña

**Affiliations:** ^1^Molecular Physiology of Behavior Laboratory, Department of Molecular, Cellular and Developmental Neurobiology, Cajal Institute, Spanish National Research Council (CSIC), Madrid, Spain; ^2^Department of Psychiatry and Behavioral Sciences, Sylvester Comprehensive Cancer Center, University of Miami Miller School of Medicine, Miami, FL, United States; ^3^Department of Neuroscience, Center for Translational Research in Neurodegenerative Disease, Center for Smell and Taste, McKnight Brain Institute, University of Florida, Gainesville, FL, United States

**Keywords:** nature, nurture, gene expression changes, behavior, neurons, epigenetics

Theories, more often than facts, are subjected to rigorous debate. The past 200 years have witnessed a most vigorous and persistent battle concerning the significance of the phenotypic diversity that, without exception, every organism exhibits. During the initial stages of biological inquiry, the significance of phenotype variability lead to the classification of living forms into distinct species and, consequently, raised the issue of whether internal or external forces were responsible for fomenting organismal speciation. Spanning from Aristotle to Darwin, over two millennia of observations elapsed until the discovery of the mechanisms of inheritance provided a solid foundation to the theory on the origin of species, by natural selection acting on the variability of reproductive populations, and birthed the most successful marriage in the history of Biology: Evolution and Genetics.

The initial matter of interest, the origin of morphological differences, has morphed into a new debate, the origin of behavioral differences. Admitted, although with reluctance, that some behavioral traits have a genetic origin, a fraction of scientists maintain today that most behaviors are learnt, and consequently, not inheritable. The, so called, “Nature vs. Nurture” debate has substituted the previous, “Evolution vs. Creation” dispute. Even though the process of natural selection, a fact rather than a theory, is hardly disputed today, one can rightfully question if the random genetic variation is the single and sufficient source of diversity upon which natural selection of behaviors could act. As the mechanisms that regulate gene expression have been progressively unveiled, it has become evident that regulatory signals can originate from inside the organism, as well as from the environment. This signaling, however, is by no means random, since it shows specificity on the genes and on the cells it targets. That is, the genome has adapted its functional structure to incorporate certain features from the environment into its normal physiology. In turn, the external manifestation of genome activity, behavior, modifies the environment for the benefit of the organism. Thus, all living bodies modify their environment, including the behavior of their own community, by their mere existence.

Despite the “Nature vs. Nurture” debate being routinely rendered defunct, in reality this debate has either been substituted by the gene regulatory puzzle in the field of biology (Traynor and Singleton, 2010), or persistently maintained in fields such as linguistics (Bowling, 2017; Kirby, 2017), art (Chirico et al., 2021) or education (Sabatello et al., 2021). Thus, the debate is still far from settled. The purpose of this special issue of Frontiers is to contribute to the visibility of emerging evidence on the bidirectional influences that link behavior and gene expression. The number of examples included here is, necessarily, very limited, but we trust that further editorial initiatives will help to publicize this growing body of data; in particular, the mechanisms that mediate the transfer of the environmental signaling from the somatic to the germinal cell lineages. The eight articles included here convey three principal messages: (1) The environment induces changes in gene expression and behavior, (2) The changes are gene and neuron specific, and (3) Behavioral adaptation also implies gene expression changes, including epigenetic modifications.

The formation and retrieval of memories was once thought to be separate and independent from genetic regulation. Reflecting the transient nature of these theories, we have here a review of the recent literature illustrating the rapid, extensive and brief wave of transcriptional activity that occurs in specific subsets of neurons as the process of memory formation develops (Roselli et al.). Noticeably, increasing evidence shows that mRNA translation and protein deployment are synapse specific, a feature that invites to reconsider what is the functional unit in the nervous system; the neuron or the synapse? Paramount examples of behavioral effects on gene expression include diet and drug-taking. Two reports address these issues and illustrate the transcriptional impact of nicotine-taking with feedback effects on addictive behavior (Sherafat et al.). Likewise, diet composition of sugar and saturated fats cause significant changes in multiple behaviors, as a comparative review of three model organisms shows here (Sarangi and Dus). Chronic exposure to certain environmental factors may cause behavioral adaptation. Alcohol intake is a classical example, and here we find an article describing the systematic identification of gene expression changes that mediate behavioral adaptation to alcohol exposure (Anqueira-González et al.). Some of these changes include epigenetic modifications at specific loci. These marks are likely to gain further relevance, either directly or indirectly, in the context of the heritability of behavior-triggered changes in gene expression.

Some types of chronic exposure do not adapt to a steady state and, in fact, evolve into a pattern of tolerance and loss of appetitive control, placing an individual at risk for addiction. Indeed, identifying the transcriptional underpinnings of addictive disorders may shed light into novel therapeutic strategies for these diseases. Consistent with this view, one of the articles from the collection shows how melatonin can lessen the opioid dependent behavioral alterations by eliciting transcriptional changes of specific genes (Alghamdi and Alshehri). Specificity is also required in the cells that build a sensory perception prior to a behavioral response. For example, the visuospatial encoding of an object to which we have to react. Two articles characterize specific areas in the peri- and post-rhinal cortices when they preprocess the vision of an object, its scale dimensions mostly, before spatial learning is completed in the hippocampus (Sethumadhavan, Hoang et al.; Sethumadhavan, Strauch et al.). This case illustrates the role of immediate early genes in the initiation of the cascade of gene expression changes. Another class of molecules with a potential significance in the relationship between the regulation of gene expression and behavior is the non-coding RNA family. One report here deals with the correlation between high levels of a specific circular RNA and the cognitive dysfunction that some elderly patients exhibit following surgical anesthesia (Zhou et al.). Circular RNAs titrate both the levels of multiple miRNAs, another type of non-coding RNA, and those of specific sets of signaling proteins. This wide range of regulatory RNAs, plus their frequent location in exosomes, anticipate their significant role in the inheritance of behavior-driven genetic effects.

In the long run, we must realize that the debate of “Nature vs. Nurture” is unjustified for the simple reason that both contenders are bound by mutual influences and, above all, they have never existed in isolation from one another.

## Author Contributions

AF: conceptualization and writing—original draft. FM, LT, and AM-P: writing—original draft. All authors contributed to the article and approved the submitted version.

## Funding

This work was supported by Spanish MICINN (Grant Number PGC2018-094630-B-I00 to FM) and NIDA grants (K01DA045294 and DP1DA051858 to LT). FM was a recipient of a RyC-2014-14961 contract.

## Conflict of Interest

The authors declare that the research was conducted in the absence of any commercial or financial relationships that could be construed as a potential conflict of interest.

## Publisher's Note

All claims expressed in this article are solely those of the authors and do not necessarily represent those of their affiliated organizations, or those of the publisher, the editors and the reviewers. Any product that may be evaluated in this article, or claim that may be made by its manufacturer, is not guaranteed or endorsed by the publisher.
